# Comprehending the Proteomic Landscape of Ovarian Cancer: A Road to the Discovery of Disease Biomarkers

**DOI:** 10.3390/proteomes9020025

**Published:** 2021-05-25

**Authors:** Shuvolina Mukherjee, Karin Sundfeldt, Carl A. K. Borrebaeck, Magnus E. Jakobsson

**Affiliations:** 1 Department of Immunotechnology, Lund University, 22100 Lund, Sweden; shuvolina.mukherjee@immun.lth.se (S.M.); carl.borrebaeck@immun.lth.se (C.A.K.B.); 2 Sahlgrenska Center for Cancer Research, Department of Obstetrics and Gynecology, Sahlgrenska Academy, University of Gothenburg, 40530 Gothenburg, Sweden; karin.sundfeldt@obgyn.gu.se

**Keywords:** ovarian cancer, proteomics, biomarkers

## Abstract

Despite recent technological advancements allowing the characterization of cancers at a molecular level along with biomarkers for cancer diagnosis, the management of ovarian cancers (OC) remains challenging. Proteins assume functions encoded by the genome and the complete set of proteins, termed the proteome, reflects the health state. Comprehending the circulatory proteomic profiles for OC subtypes, therefore, has the potential to reveal biomarkers with clinical utility concerning early diagnosis or to predict response to specific therapies. Furthermore, characterization of the proteomic landscape of tumor-derived tissue, cell lines, and PDX models has led to the molecular stratification of patient groups, with implications for personalized therapy and management of drug resistance. Here, we review single and multiple marker panels that have been identified through proteomic investigations of patient sera, effusions, and other biospecimens. We discuss their clinical utility and implementation into clinical practice.

## 1. Introduction

Ovarian cancer (OC) is often used as an umbrella term referring to malignancies caused by ovarian epithelial inclusion cysts that are trapped beneath the surface of the epithelium of the ovary as well as malignancies in the peritoneum and fallopian tube [1]. Advanced OC is one of the deadliest malignancies in women with a 5-year survival rate below 30% and high incidences of occurrence in the Eastern and Central European population (11.4 per 100,000 and 6.0 per 100,000, respectively) [2]. Although the incidence varies across populations, the average lifetime risk of developing OC is 1.3% [3].

Most OC are epithelial (90%), and it is a heterogeneous disease comprising of a range of subtypes [4]. The most frequent subtype is high-grade serous carcinoma (HGSC) corresponding to around 60 % of cases, whereas low-grade serous carcinoma, mucinous, clear cell, and endometrioid OC are all less abundant [4]. The spread of OC is frequently systematically categorized using a scoring scheme outlined by the International Federation of Gynecology and Obstetrics (FIGO). FIGO scoring is based on the tumor-node-metastasis (T-N-M) approach which systematically describes the extent of the tumor (T) as well as its spread to lymph nodes (N) and potential metastasis (M) and categorizes OC into 4 stages (denoted I, II, III, and IV). Stage I is characterized OC only in the ovary(s) or fallopian tube(s) and Stage II by its spread to a close organ such as the uterus, bladder, or rectum. Stage III is defined by the spread to the abdomen and/or lymph nodes and stage IV by distant metastasis. i.e., pleura. While Stage I tumors are associated with a good prognosis most OC cases are not diagnosed at this stage. Stage II and III OCs are removed by debulking surgery followed by treatment with a combination of platinum and taxane chemotherapy which leads to considerable improvement in survival [5]. Stage III tumors are categorized by the spread to the adjacent peritoneum through metastasis. Stage IV is defined through distant metastasis and frequently treated by a combination of debulking surgery to remove the primary tumor and chemotherapy to target metastases. Due to the lack of efficient tools for early diagnosis, around 10–20% of the OCs are detected at this stage and treatment options remain limited along with poor survival rates [6]. OC tumors are typically also categorized as low or high grade, which reflects the differentiation state of tumor cells. The less differentiated low-grade tumors are typically associated with a better prognosis.Several genetic studies have linked dysregulated gene expression and mutations to OC. However, not all OCs display a similar pattern, emphasizing that the disease is heterogeneous also at the molecular level. For example, and by analogy with malignant breast cancer, mutations in BRCA1 and BRCA2 are linked to OC [7]. Moreover, the high-grade serous OCs display a high frequency of TP53 mutations and other OC histologic subgroups have frequent mutations in ARID1A, PIK3CA, PTEN, CTNNB1, KRAS, and RPL22 [7,8,9,10].

One of the major challenges associated with the diagnosis of OC is the asymptomatic nature of the disease. Early-stage (I and II) OC are therefore challenging to detect. Late-stage (III and IV) OC is associated with more severe symptoms, and invasive surgery is the most viable option for disease management [11]. Although primary complete debulking surgery (PDS) strikingly increases survival for advanced-stage OC, it is not a perfect approach and many patients suffer from the recurring disease. In certain cases, the tumor burden needs to be reduced before PDS. This is frequently achieved through neo-adjuvant chemotherapy and referred to as interval debulking surgery [12].

**Figure 1:**
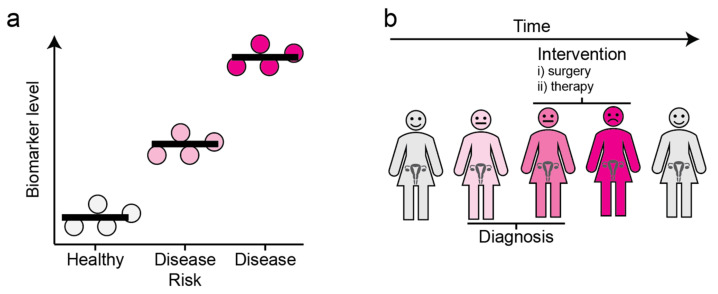
The concept and utility of biomarkers. (**a**) Disease biomarkers. Molecules of which the level is associated with a disease state are referred to as biomarkers. (**b**) Clinical utility of biomarkers. The levels of biomarkers can be monitored over time, allowing for early diagnosis and informed decisions regarding clinical interventions. Grey: Healthy, Light Pink: Individuals with disease risk, Dark Pink: Individuals harboring the disease.

While the 5-year survival rates for early-stage (I and II) OC can be up to 90% with clinical interventions like cytoreductive surgery and combination chemotherapy, the late-stage (III and IV) OC 5-year survival rate is below 30% [13]. Therefore, diagnostic biomarkers that distinguish benign from malignant tumors at an early stage would be of tremendous value (Figure 1a). Moreover, as OC is a complex heterogeneous disease, biomarkers predicting the responsiveness of tumors to drugs, which would thereby guide personalized treatment, would be of great clinical utility (Figure 1b).

## 2. Protein Biomarkers Associated with OC

**Figure 2:**
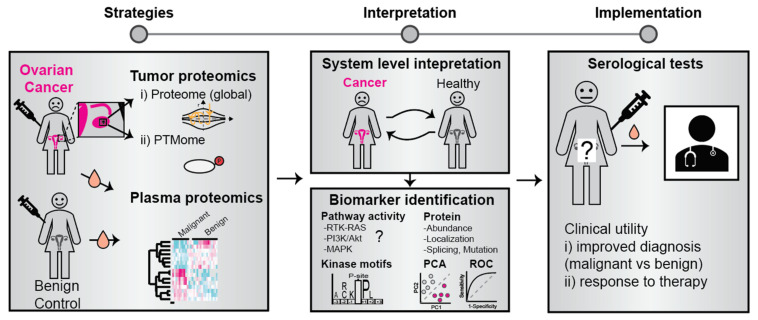
Identification and clinical use of OC protein biomarkers. Biomarkers can be identified by a comparative analysis of proteins and their modification state in tumor material and blood plasma from patients and controls. The bioinformatic analysis may involve cellular pathway activity mapping, principal component analysis (PCA), and receiver operator characteristics (ROC). Identified biomarkers have the potential to improve disease diagnosis and predict response to therapy.

The identification of biomarkers for improved OC diagnosis and informed clinical decision-making would represent great value for both patients and the healthcare system. Protein markers are most frequently analyzed in the tumor, tumor effusions, or circulating fluids such as blood plasma (Figure 2). Early studies have reported the use of single markers in blood serum such as CA125 (Uniprot ID Q8WXI7, also known as Mucin-16) [14] and HE4 (Uniprot ID Q14508) [14]. With high-throughput semi-automated systems for sample handling and analysis, as well as the implementation of machine learning-based AI approaches, the use of biomarker panels comprising multiple markers has emerged as a superior approach. For example, panels of analytes such as the one proposed by Mor G et al., consisting of Leptin, Prolactin, Osteopontin, and insulin-like growth factor-II (IGF-II) have been proven to be useful for discriminating cancer and non-cancer patients as well as the assessment of stage I/II disease [15]. In a recent study, the assessment of multiple biomarkers, along with CA125, considerably improved the performance of the predictive model for early diagnosis. This panel comprised of CA125, HE4, CHI3L1, PEBP4, and/or AGR2, provided 85.7% sensitivity at 95.4% specificity up to one-year before diagnosis [16]. Moreover, a study by Enroth et al., recently revealed a candidate 11-protein biomarker panel for early OC diagnostics [17]. Currently, studies aimed at uncovering OC biomarkers are increasingly implementing similar multiple-marker models for predictive analysis (Table 1).

Proteomic characterization of tumor tissue specimens has also revealed molecular aberrations that contribute to the onset and progression of OC [28,29]. Immunohistochemistry-based examination of tumor specimens using members of the cytokeratin family (CK7 and CK20) helps in distinguishing serous OC from other gastrointestinal malignancies [30]. In-depth analysis of genetic and histopathological signatures has also led to categorizing OCs into two types, Type I (Low grade) and Type II (High grade). While Type I tumors have a high frequency of KRAS and BRAF mutation, Type II tumors have a high frequency of TP53 mutations [31,32,33,34]. Other biospecimens such as effusions, pap smear fluids, and cervical swabs, are also valuable for understanding OC pathobiology and represent sources of markers that can predict clinical outcomes [35,36]. For example, a 9-biomarker panel in ovarian cyst fluids has been shown to discriminate between type 1 and type 2 tumors [37]. Moreover, a pilot study has depicted the utility of vaginal lysophosphatidic acid (LPA) levels as a non-invasive diagnostic marker for OC in post-menopausal women [38]. Another study investigating OC effusions revealed prominent involvement of cell-cell adhesion molecules like FAK, Erk, and P-Cadherin [39]. The study also suggested that cell adhesion molecules can comprise a prognostic signature that can be utilized to predict tumor aggressiveness as well as patient segregation. Cell adhesion protein expression, when correlated with clinicopathological parameters, has also been used to identify patient cohorts for clinical trials with small molecule inhibitors of FAK and other upstream effectors [40,41,42]. While these markers have yielded insights into OC development and the molecular pathways associated with it, they are still in the early stages of investigation and are yet to be implemented for disease management.

The emergence of ‘liquid biopsies’ has indeed ushered in a new era in diagnostics [43]. There is now tremendous potential for identifying biomarkers for improved OC diagnosis by mining such liquid biopsies with state-of-the-art (prote)-omics technologies [44,45]. Mostly the liquid biopsies are probed for circulating tumor DNA, tumor cells, exosomes, or tumor microRNA. In OCs, circulating tumor cells (CTC) are often present and useful as surrogate markers of minimal residual disease. In a study by Zhang et al., wherein nearly 100 patients were screened and subjected to CA125 measurements; CTCs were detected in nearly 90% of the newly diagnosed patients. The number of CTC also correlated with the stage of the OC. However, the ratio of CTC in comparison to other components in plasma is low and the choice of detection technique influences the number of CTCs identified. Although major strides have been made through the implementation of liquid biopsies for several cancers, more research is required to assess the full utility of CTC determination for OC, which primarily metastasizes directly through the abdominal cavity [46,47,48,49,50].

With recent advances in high-throughput omics technology and automated handling of large sample cohorts, the scope of establishing multi-marker panels has increased considerably. An ideal scenario for the effective clinical management of OCs would implement an integrated approach where blood-based markers and imaging analysis are collectively used for diagnosis and guiding clinical decisions on surgery and choice of therapy.

## 3. Candidate Markers for OC Diagnosis in Circulatory Fluids

CA125 (Uniprot ID Q8WXI7) is a large glycoprotein with a molecular weight of over 1.5 M Da, and is one of the most extensively used tumor markers for OC. Its link to OC was first described in 1981 by Bast RC et al., and its level is elevated in 90% of high-grade OC patients [13,51]. CA125 is considered a ‘gold standard’ marker for OC and is widely used for clinical assessment regarding disease progression and therapeutic efficacy in OC patients [51]. However, its utility in early diagnosis is limited as its expression is only found to be elevated in the later stages of OC and is often found to be elevated even in cases of benign endometriosis [52,53]. Therefore, the quest for identifying markers that can aid in early detection remains an area of active research.

A series of other markers have subsequently been identified from OC patient sera, including the Human epididymis protein 4 (HE4) (Uniprot ID Q14508), which is a secreted glycoprotein that is overexpressed in both serous and endometrioid OCs and thus might be useful in specific clinical scenarios [45,54,55]. In the same vein, line protein markers like EGFR, ErbB2, and osteopontin, in combination with the above-mentioned, have been reported to be of relevance in OC. These markers exhibit greater sensitivity and specificity when compared to single biomarker assays for the early detection of OCs [56,57]. However, the utility of these markers in isolation and combination remains a challenge when it comes to early diagnosis of the advent of ovarian malignancy [1].

The development of multivariate index assays (MIA) that comprise panels of biomarkers to assess the extent of malignancy has greatly facilitated the identification markers that can also aid in early diagnosis. For example, the FDA-approved MIA test Ova1^®^ is based on a 5-protein biomarker panel (CA125, TF, B2M, Transthyretin, APOA1) that serves to aid clinical decision-making regarding surgery in the case of ovarian adnexal mass [8,58]. Similarly, the ROMA^®^ test comprises two proteins (CA1254 and HE4) that predict the risk of finding a malignancy during surgery in the case of an increased ovarian adnexal mass, but the ROMA test has established predictive power for both pre-and postmenopausal women [1,52,53,59]. The above-mentioned panels highlight the utility of plasma biomarkers for clinical assessment of OCs, and add confidence to the prospects of taking markers identified in proteomics experiments from bench side to bedside. Notably, none of the approved tests have yet shown utility for screening purposes [60,61].

Blood is the preferred source of biomarkers as it is routinely extracted and handled at primary care units and hospitals. Therefore, blood-based biomarkers can be readily assessed which is a considerable advantage over biospecimens extracted with invasive methods, such as tumor biopsies or effusions. Consequently, a large number of clinical studies have been undertaken using proteomics platforms to assess both diagnostic and prognostic markers from patient sera and plasma [62,63]. Investigation of serum proteome using mass spectrometry platforms has led to the identification of many differential markers including the three- biomarker panel consisting of APOA1, transthyretin (downregulated), and inter-*α*-trypsin inhibitor heavy chain H4 (cleavage fragment) (upregulated) as well as CTAPIII and PF4 [26,64,65,66] (Table 1). Many of these markers are now being screened using highly sensitive targeted mass spectrometry-based methods such as selected reaction monitoring (SRM) [14]. A recent study using an SRM assay was able to identify a 5 protein panel consisting of IGHG2, LGALS3BP, DSG2, L1CAM, and THBS1 which yielded almost 94% specificity, along with CA125, for distinguishing OC patients from healthy controls [67]. These developments highlight the potential of integrating multi-protein panels identified by mass spectrometry studies into mainstream clinical diagnostics modules.

## 4. Proteomic Profiling of Solid Tumors and Clinically Relevant Protein Markers

Recent advances in mass spectrometry-based workflows have contributed to the identification of several proteins linked to OC in both tumor biospecimens as well as in plasma and serum [68,69,70]. These studies have employed a plethora of strategies that include label-free data-dependent acquisition (DDA), label-free SWATH, and isobaric protein labeling approaches denoted TMT [37] or iTRAQ [70,71]. This has resulted in the identification of several proteins that previously had not been well studied in the context of OCs. This includes transtherytin (TTR), apolipoprotein A1 (APOA1), and casein kinase II alpha 1 subunit isoform-a (CSNK2A1), as well as markers indicative of drug resistance such as Destrin (DSTN), Tumor rejection antigen (gp96) (HSP90B1), and EGF-containing fibulin-like extracellular matrix protein 1 (EFEMP1) [62]. These markers were shown to correlate with the clinical outcomes of the tumor. For instance, elevated levels of glycoprotein tumor rejection antigen (gp96) were observed in a human OC cell line that was resistant to paclitaxel vs nonresistant OC cell lines [72]. Similarly, HGSCs, which account for around 70–80% of morbidities, often exhibit resistance to conventional chemotherapy. System-wide analyses of both genomic and proteomic evidence have revealed that inhibition of PI3K/AKT/mTOR pathway components can be an alternative approach for patient management [73]. Quantitative proteomics studies on cancer cell lines as well as more sophisticated approaches using PDX models are pivotal in uncovering cancer cell vulnerabilities that can then be targeted by specific inhibitors for efficient management of aggressive tumors [73,74,75]. The proteomic profiling of tumor tissue has also revealed widespread aberration in key cellular pathways such as the p38-MAPK stress-activated signaling pathway and cytoskeletal components [76]. A dysregulated pathway can represent a tumor vulnerability and is key in designing therapeutic intervention strategies. In a recognized initiative by the Clinical Proteomic Tumor Analysis Consortium (CPTAC), a comprehensive study of nearly 100 patients with HGSC was performed along with 25 controls consisting of normal tissues. Using both label-free and isobaric labeling quantitative proteomics approaches to this dataset has enabled a deeper understanding of the protein level alterations in HGSC. For example, the proteomic characterization linked post-translational modifications (PTM), including phosphorylation and glycosylation, to OC progression. The CPTAC data have also enhanced our understanding of OC heterogeneity and enabled the classification of HGSC subgroups based on gene changes using the TCGA database and proteome composition from the CPTAC data. Further investigation revealed a group of kinases that had elevated activity in tumors, including several mitotic kinases, AURKA and cyclin dependent kinases, CDK1, CDK4, and CDK7. These represent potential intervention points for HGSC therapy and can pave the way for better treatment regimens [76,77,78]. Ultimately, the deep proteomic profiling of the tumor tissues has enabled the subtyping of tumors and the prediction of overall survival of HGSCs. For instance, the global proteomic profile of the HGSCs corresponded to TCGA transcriptomic subtypes, namely the mesenchymal, proliferative, immunoreactive, and differentiative [69]. Tong et al. has further extrapolated the data from the CPTAC to identify subtypes based on the patient phosphoproteome, as well as key druggable kinases. The activity of eight kinases including CDK9, CDC7, BRAF, and AXL, which were part of the Ph4 and Ph5 subtypes, also correlated with patient survival [79]. Collectively, these studies have pin-pointed clinically actionable targets.

## 5. Identification of PTMs in Ovarian Cancer and Their Clinical Implications

Proteins are frequently modified by so-called PTM, examples of which include glycosylation, phosphorylation, and methylation, as a means to regulate and tune their function [80,81]. All PTMs involve an increase in the mass of the modified protein and are almost invariably analyzed in scale using mass spectrometry-based workflows [82]. The identification of several high molecular glycoproteins associated with OC like CA-125 and HE4 suggests a link between glycosylation and the disease pathobiology. Indeed, glycoproteomic profiling of high-grade serous ovarian carcinomas (HGSC) has revealed differential glycosylation patterns between OC subtypes. This can aid in patient stratification and promote the understanding of the role of glycosylation in tumor etiology and development [72]. Therefore, the implementation of comprehensive glycoproteomic profiling has great potential in future OC biomarker studies.

A recent study that employed high-density HuProt™ arrays and used a multiplexed PTM analysis approach, revealed several dysregulated signaling proteins, including members of the Src kinase family and focal adhesion kinases, in HGSC. An in-depth analysis revealed prominent alterations of key PTMs, including tyrosine phosphorylation, and identified key kinases such as SYK and PTK2B that represent potential drug targets [83].

Another prominent PTM linked to OC is the site-specific methylation of 70 kDa heat shock protein (Hsp70) at Lys561 [84]. The site is specifically and exclusively modified by the enzyme METTL21A [85,86] as a means to modulate the chaperone activity of Hsp70 [87], and loss of methylation at the site has been linked to poor prognosis and reduced overall survival [84]. Notably, Hsp70-K561 methylation constitutes a part of the so-called ‘chaperone code’ [88,89], where multiple PTMs, individually or collectively, act to regulate chaperone function. Taken together, this suggests that precise regulation and PTM-mediated tuning of Hsp70 activity is important in OC biology.

A deep proteomic investigation on patient-derived primary cell lines from epithelial OC revealed that there are differentially expressed proteins as well as phosphorylation sites that can discriminate between cancer and healthy cells. In detail, the phosphorylating kinase enzyme CDK7 was identified as a druggable target, representing a novel therapeutic entry point and strategy [90].

Collectively, these recent insights from proteomic characterization of PTMs in patient biospecimens and cancer cell lines highlight the value of analyzing protein modifications, in addition to protein levels, in biomarker studies (Table 2). As PTM proteomics technology develops, we anticipate an increased focus on such studies. Protein PTM status will likely represent a key feature in next-generation OC diagnostics.

## 6. Ovarian Cancer Drug Resistance and Proteomics

The standard method of care for OC involves surgery and chemotherapy with carboplatin and paclitaxel [93]. However, drug resistance is a major problem for many patients. Proteomic investigations have attempted to decipher the underlying mechanism of drug resistance in the patient cohorts and have indicated aberrations in the ATP synthesis and RAN GTPase binding components [94]. Moreover, analysis of serum samples of OC patients has suggested the involvement of FN1, SERPINA1, and ORM1 [95]. These markers may potentially be used as a panel to identify resistant patients prior to chemotherapy (See also Table 2). Other studies link aberration of key signaling cascades such as PI3K/Akt/mTOR pathway to OC drug resistance [96,97,98,99]. Overexpression of EGFR and subsequent EGFR-mediated angiogenesis and further metastasis is also very common among high-grade patients [100,101].

It is clear that OC can obtain resistance through a range of pathways, many of which are linked to oncogenic kinase signaling [102]. Recent advances in single-cell technologies such as spatial profiling [103] and single-cell proteomics [104] have great potential for uncovering tumor heterogeneity and shedding light on mechanisms for inherent and acquired drug resistance. Mechanisms underlying drug resistance can be delineated using preclinical OC cell line models [105,106]. We envision that this will also be a focal point for OC research in the coming decade.

## 7. High-Density Protein Microarrays and OC Biomarkers

The development and utility of high-density protein microarrays have led to significant progress in the field of cancer proteomics [14,107]. For example, human proteome arrays comprising nearly 17000 full-length proteins in duplicate have enabled studies uncovering specific and differential autoantibody responses between OC patients and controls [108,109]. Using this full-length protein array technology, studies have found that markers such as Lamin A/C, SSRP1, and RALBP1 are elevated in OC patients [110]. Antibody microarrays can also be useful for screening disease-associated markers based on the specificity of the antigen-antibody interactions. Using high-density arrays with immobilized antibodies provides a rapid approach to screen for cancer-specific markers from complex proteomes, such as human serum [107,111]. Antibody-microarrays have developed to the point where very high test accuracy can be achieved, as is illustrated by the detection of early-stage pancreatic cancer [112]. This technology also provides a way to validate markers for clinical utility identified by orthogonal techniques, such as mass spectrometry.

## 8. Perspective on Existing Biomarker Panels: Their Utility, Limitations, and Future Scope

The journey of identifying markers for early diagnosis of OCs, from CA125 & HE4 to current multi-marker models, has indeed come a long way [107]. Notable progress has been made in uncovering molecular features and mechanisms of the heterogeneous malignancy. Several studies have shown that multivariate biomarker panels outperform single marker analysis. For example, the 6 marker panels comprising leptin, prolactin, osteopontin, insulin-like growth factor 2, macrophage inhibitory factor, and CA125 outperformed CA125 alone, with a specificity of 99.4% [14,56]. Many biomarkers display good performance in single cohorts, but most fail in independent validation studies [113]. To cope with this, a recent effort by the Mann Lab systematically identified plasma proteins related to sample handling bias to generate a resource of “low quality” markers [114].

Recently, Enroth et al., described an 11-protein biomarker signature that separated ovarian cancer stages I–IV from benign controls with a specificity of 93% in an independent validation cohort [17]. However, no report has so far demonstrated high sensitivity for early-stage OC. Current challenges include increasing the specificity of the multi-marker panels, as well as the establishment of robust technological platforms that can easily quantify these markers from patients in a clinical setting.

The development and clinical implementation of complex multivariate algorithms that combine age, menopausal status, imaging, and serum-based biomarkers into a single index for estimation of the risk of malignancy have benefitted patients. For example, strategies such as Risk of Malignancy Index (RMI) [115], Risk of Malignancy Algorithm [20] (ROMA), and OVA1 [20] have been useful to aid decisions regarding treatment strategy, and specifically whether to refer the case to specialized cancer surgery in the case of increased pelvic mass [116,117]. Such multi-variate marker panels will now also be developed for early diagnosis, and there is currently a window of opportunity for high throughput (prote)-omics technologies to uncover such markers.

The biomarker diagnostic test format is a key question for clinical implementation. Today, single or multiplexed antibody-based tests, such as ELISA, represent the most frequently used method. Antibody-based tests have two clear benefits compared to MS-based platforms. First, they can readily target low abundant proteins that a mass spectrometer is typically blind to due to abundance detection bias and the vast dynamic concentration range of proteins in plasma [63]. Second, most biomedical hospital labs have the instrumentation and knowledge to perform such tests, but they rely on commercial tests based on highly specific antibodies. In that respect, MS is a more versatile method that allows the targeting of any protein, although it is still a niche field and few clinics are equipped with the instrumentation and expertise required for its conduction. For MS to be widely applied, the establishment of tailored reference laboratories that are dedicated to such analysis would be a solution. Hospitals would then send samples to be analyzed in such expert biomarker laboratories, a logistic procedure most hospitals have already implemented.

## 9. Conclusions and Outlook

Circulating protein biomarkers display great potential to discriminate between patients with benign and malignant ovarian cysts, while also guiding treatment decisions [8,118]. In recent years, proteomics characterization of plasma, effusions, and solid tumors has uncovered molecular mechanisms and a plethora of candidate biomarkers for OC, although these still need to be validated to show clinical utility. We foresee a wealth of studies in the coming years validating these candidate markers, while also identifying additional markers. We also anticipate that basic OC research will focus on the single-cell resolved analysis of tumor protein and PTMs. The integrated analysis of tumor specimens with matched blood samples is particularly interesting and has the potential to reveal accessible surrogate blood-based biomarkers that reflect tumor biology and can be used in personalized treatment.

## Figures and Tables

**Table 1:** Examples of key protein markers associated with Ovarian Cancer.

Marker(s)	Gene ID (If Applicable)	Source	Type (Circulatory/Tumor-Specific	Utility (Early/Late-Stage Pre/Post-Menopausal)	Platform & Study Design	Reference
CA-125	MUC16	Serum/Plasma	Serum marker-high molecular weight glycoprotein	Monitoring response to chemotherapy and disease activity in clinical trials.	Immunoassays from patient sera using OC125 and M11 antibodies	[18,19,20,21]
HE4	WFDC2	Serum/Plasma	HE4 is also a secreted glycoprotein that is overexpressed in OCs	FDA approved biomarker for monitoring disease activity	Immunoassays from patient sera	[19,20]
MCSF and LPA	CSF1	Blood/Tumor tissue ascites	Components of the tumor microenvironment	LPA is elevated in the blood, tumor tissue, and ascites. LPA also influences tumor-associated macrophages, which can be used as a therapeutic target	Metanalysis from several studies mostly based on the immunoassay-based determination of markers	[22]
CART analysis: CA-125, OVX1, LASA, CA 15-3, CA 72-4)	MUC16, ovx1, MUC1	Serum	Circulatory markers as well as tumor microenvironment components	CART analysis (classification and regression tree analysis), uses the sequential analysis of marker concentrations with 5 markers (CA-125, OVX1, LASA, CA 15-3, CA 72-4) to yield a sensitivity of 90.6% and a specificity of 93.2%	Initial discovery-based studies using radioimmunoassay. Multiple marker analysis performed on ANN based machine learning algorithms	[23,24,25]
A three-panel marker: Apolipoprotein I TransthyretinInter-α-trypsin inhibitor heavy chain H4 (cleavage fragment)	APOA1, TTR, ITIH4	Serum	Components of the circulatory biofluids	Useful for detection of early-stage patients, exhibits higher sensitivity (74%) over CA125 alone (52%)	The study employed SELDI-TOF technology with the ProteinChip Biomarker System (Ciphergen Biosystems)	[25,26]
CT45	CT45A1, CT45A	Tumor tissue (FFPE blocks)	Tumor marker	Reported to be an independent prognostic factor that is associated with a doubling of disease-free survival in advanced-stage HGSCs	Quantitative proteomics on FFPE tumor samples derived from 25 chemotherapy-naive patients with advanced-stage HGSCs	[27]
MUCIN-16, SPINT1, TACSTD2, CLEC6A, ICOSLG, MSMB, PROK1, CDH3, WFDC2, KRT19, and FR-alpha	MUCIN-16, SPINT1, TACSTD2, CLEC6A, ICOSLG, MSMB, PROK1, CDH3, WFDC2, KRT19, and FOLR	Plasma	Circulatory markers	Potentially useful for improved diagnosis of adnexal ovarian mass and identification of potential cases for specialized referrals	PEA was implemented utilizing oligonucleotide antibody probes to measure protein abundance	[17]

CA-125 = cancer antigen 125; HE4 = homo sapiens epididymis specific 4; MCSF = macrophage colony-stimulating factor; LPA = lysophosphatidic acids; ANN = artificial neural networking; SELDI-TOF = surface-enhanced laser desorption/ionization-time of flight; HGSC = high-grade serous ovarian carcinomas; PEA = proximity extension assay.

**Table 2:** Example biomarkers linked to PTM and drug resistance in OC.

Marker(s)	Source	PTM Details/Drug Resistance/Other	Platform	Reference
FAK, PTK2B	Ovarian cell lines	Phosphorylated	Protein microarrays: HuProt arrays	[83]
POSTN, SERPINA1, HYO1	HGSC tumor tissues	Glycosylation	SPEG for glycosite analysis & intact glycopeptides for investigation of IGPs followed by LC MS/MS	[72]
TGFBI, OPN	Ovarian cell lines	Drug resistance against cisplatin and paclitaxel	Protein microarray: Affymetrix GeneChip Human Genome U219 microarrays	[91]
COL5A2, LPL	Exosomes derived from normal human ovarian surface & cancer cell line	Elevated levels seen in exosomes derived from cancer cells	Exosome isolation followed by LC MS/MS	[92]
HSPA1 (Hsp70)	Tumor effusions from HGSCs	Methylation status of Lys561	LC MS/MS analysis	[84]

SPEG = solid-phase extraction of glycosite-containing peptides; IGPs = glycosite-specific glycans.
